# Effects Of Early Trauma and Recent Stressors on Depression, Anxiety, and Anger

**DOI:** 10.3389/fpsyt.2020.00744

**Published:** 2020-08-04

**Authors:** Bum Joon Seok, Sehyun Jeon, Jooyoung Lee, Seong-Jin Cho, Yu Jin Lee, Seog Ju Kim

**Affiliations:** ^1^ Department of Psychiatry, Samsung Medical Center, Seoul, South Korea; ^2^ Department of Psychiatry, Korean University Anam Hospital, Seoul, South Korea; ^3^ Department of Psychiatry, Gachon University Gil Medical Center, Incheon, South Korea; ^4^ Department of Psychiatry, Seoul National University College of Medicine, Seoul, South Korea; ^5^ Department of Psychiatry, Sungkyunkwan University School of Medicine, Samsung Medical Center, Seoul, South Korea

**Keywords:** depression, anxiety, anger, trauma, stress

## Abstract

**Background:**

Early life traumatic events and recent stressful events are known to have especially strong effects on emotional wellbeing. However, little is known about the interaction of early and recent stressors on emotions. We aimed to examine the interactive effects of early trauma and recent stressors on depression, anxiety, and anger.

**Methods:**

One hundred and seventy adults were recruited and asked to complete the Center for Epidemiological Studies Depression Scale (CES-D), the state anxiety subscale of the State–Trait Anxiety Inventory (STAI-S), and the state anger subscale of the State–Trait Anger Expression Inventory (STAXI-S). Early traumas and recent stressors were assessed during face-to-face interviews. Multiple regression analysis was performed to test whether early trauma, recent stressors, and the interaction of the two would predict CES-D, STAI-S, and STAXI-S scores.

**Results:**

In the multiple regression models, STAI-S scores were predicted only by recent stressors (*R^2^* = 0.063, *p =* 0.001). In contrast, CES-D and STAXI-S scores were predicted only by the synergistic interaction of early trauma with recent stressors (*R^2^* = 0.075, *p <* 0.001; *R^2^* = 0.039, *p =* 0.01, respectively).

**Conclusions:**

A synergistic interaction effect between early trauma and recent stressful events on current depression and anger was observed, indicating that the combined effects of early trauma and recent stressors are stronger than their individual effects. In contrast, anxiety was affected mainly by recent stressors. Our findings suggest that the form that emotional disturbance takes can vary depending on the timing of stressors.

## Introduction

Stressful life events can contribute to various emotional disturbances such as depression, anxiety, and anger (1–4). Stressful life events seem to affect emotion *via* various mechanisms, including variations in neurological development, epigenetic modifications, neuroendocrine modulation, or changes in brain circuits (5–12). Recent stressful life events have been suggested to stimulate conditions for subsequent depression, anxiety, or anger (1, 13–17). In addition, early traumatic events are known to have strong, long-term effects on emotional well-being (18, 19), being associated depression, anxiety (20–22), and anger (23, 24). A previous study reported that childhood adversity leads to higher levels of anger and aggression (23). Childhood adversity and anger have also been reported to be associated with neurobiological changes, such as altered activity in the amygdala and dorsolateral prefrontal cortex (24). Recent stressors can also provoke anger (4, 25). Anger is an important issue when studying the emotional effects of trauma or stress, and should be assessed separately from anxiety and depression. The effects of childhood traumatic events on emotional disturbances may continue into adulthood (26).

As stressful events have cumulative effects (27–30), the long-term effect of early trauma and the short-term effects of recent stressors may simply be additive. However, it may also be that the early trauma and recent stressors have synergistic effects on emotional responses such as depression, anxiety, and anger. Given that the neurobiological mechanism by which early life trauma affects subsequent emotion may be different from that for recent stressful life events, these two different mechanisms may have synergistic or interacting effects on the development of emotional disturbances (12).

Also from a psychological viewpoint, children with early adversity exhibit more severe emotional disturbances when they experience subsequent stressful events. Individuals who have experienced trauma during childhood may show emotional dysregulation (31). Emotional regulation plays an important role in coping with stress. Dysfunctional emotion regulation strategies, such as high expressive suppression, are associated with emotional difficulties (32). Childhood traumatic experience can be a risk factor for negative emotions through the mechanism of emotional dysregulation. Individuals with early traumatic experiences may be more likely to exhibit emotional disturbances during subsequent stressful situations (33–36). In other words, childhood traumatic experience can exacerbate the emotional disturbances caused by subsequent stressful life events (37, 38).

Several studies have investigated both early trauma and recent stressors in a single study. However, only few have explored the interactive effects of early trauma and recent stressors on depression, and those produced controversial results. One study reported the interactive effects of childhood adversity and recent life stressors on suicidal behavior among Chinese college students (39). Others reported that young Americans (37) and psychiatric outpatients in Germany (38) who reported childhood adversity became more depressed after experiencing recent stressors. However, another study found no effect of the interaction between early and recent stressors on depression in older people (26). These discrepancies may be due to differences in sample characteristics, such as age or psychiatric illness. Furthermore, no studies have investigated such interactive effects on anxiety or anger.

In the current study, we aimed to explore the effects of early trauma, recent stressors, and their interaction on depression, anxiety, and anger in a community sample covering a broad range. We hypothesized that the interaction between early trauma and recent stressors would exert a synergistic effect on subsequent depression, anxiety, and anger. In other words, early trauma is posited to exacerbate the effects of recent stressors, thus promoting emotional disturbances, while recent stressors should in turn exacerbate the effects of early trauma.

## Methods

### Subjects

Community-dwelling adults (≥19 years of age) were recruited in the Incheon area, which is the third largest metropolitan area in South Korea. Participants were recruited through posters and brochures placed in hospitals, apartment buildings, churches, and public health centers. All participants were asked to self-report their levels of depression, anxiety, and anger.

A total of 170 subjects participated in the study, including 73 men and 97 women. The mean age of subjects was 41.1 ± 8.2 years; there was no significant difference in age between men and women (**Table 1**).

**Table 1:** Comparison of depression, anxiety, and anger Between subjects with and without early trauma or recent stressors.

	ET-RS	ET-NRS	NET-RS	NET-NRS	Total	Post hoc
	(*n* = 16)	(*n* = 49)	(*n* = 24)	(*n* = 81)	(*n* = 170)	
	Mean ± S.D., *n* (%)	Mean ± S.D., *n* (%)	Mean ± S.D., *n* (%)	Mean ± S.D., *n* (%)	Mean ± S.D., *n* (%)	
Demographic variables						
Age (year)	41.9 ± 8.1	39.6 ± 7.0	41.8 ± 6.1	41.6 ± 9.4	41.1 ± 8.2	–
Gender (male)	5 (31.3%)	26 (53.1%)	9 (37.5%)	33 (40.7%)	73 (42.9%)	–
Depression						
CES-D score^*^	19.4 ± 12.8	11.7 ± 8.2	12.1 ± 10.3	10.1 ± 8.0	11.7 ± 9.2	ET-RS > ET-NRS, NET-NRS
Anxiety						
STAI-S score^*^	53.0 ± 7.6	48.7 ± 6.5	51.6 ± 8.1	46.9 ± 7.8	48.7 ± 7.7	ET-RS, NET-RS > NET-NRS
Anger						
STAXI-S score	14.8 ± 6.0	13.3 ± 4.6	13.0 ± 5.7	11.9 ± 3.3	12.7 ± 4.4	–

ET-RS, those with both early trauma and recent stressors; ET-NRS, those with early trauma but without recent stressors; NET-RS, those with recent stressors but without early trauma; NET-NRS, those with neither early trauma nor recent stressors; CES-D, Center for Epidemiological Studies-Depression Scale; STAI-S, state anxiety subscale of the Korean version of the State–Trait Anxiety Inventory; STAXI-S, state anger subscale of the Korean version of the State–Trait Anger Expression Inventory.

^*^p < 0.05 on the Kruskal–Wallis test.

The study protocol was approved by the institutional review board of Gachon University of Medicine and Science. All subjects provided written informed consent.

### Assessment of Early Trauma and Recent Stressors

Information on early trauma was collected through face-to-face interviews using the Early Life Stress Questionnaire (ELSQ). ELSQ is a questionnaire based on the Child Abuse and Trauma Scale (40). With ELSQ, responders are asked to indicate whether they experienced any of 19 negative events, such as physical and sexual abuse and neglect, in early life (41).

Information regarding recent negative stressors was also obtained through face-to-face interviews. During the interview, subjects were asked whether any of the items extracted from the Life Experience Survey (LES) described their own experience. The questions asked about (1) bereavement involving family or close friends; (2) serious illness in oneself or a family member; (3) serious economic crisis; (4) unwanted retirement or being fired; (5) severe conflicts with family, friends, relatives, or colleagues; and (6) divorce or unwanted end of a relationship (42, 43). To minimize the possibility of recall bias, participants were asked to report only experiences that had happened during the 6 months prior to their participation in the study.

All subjects were divided into four groups based on the presence of early trauma and/or recent stressors: those with both early trauma and recent stressors (ET-RS); those with early trauma but no recent stressors (ET-NRS); those with recent stressors but no early trauma (NET-RS); and those with neither early trauma nor recent stressors (NET-NRS). Subjects were also divided into two groups according to the presence/absence of early trauma and the presence/absence of recent stressors, independently of whether they had experienced the other form of stressor.

### Assessments of Emotional Disturbances

To measure depressive symptoms, the Korean version of the Center for Epidemiological Studies Depression Scale (CES-D) was used. The severity of depression is reflected in CES-D scores, with higher scores indicating more severe depression. The Korean version of the CES-D is well verified. It consists of 20 questions and has an internal reliability coefficient of 0.89. The Korean version of the CES-D tends to produce higher scores than the original version (44, 45).

The Korean version of the State–Trait Anxiety Inventory (STAI) was used to measure current anxiety. A high score indicates high levels of anxiety. STAI is divided into subscales assessing state anxiety (the current anxiety level) and trait anxiety (intrinsic emotional characteristics (46, 47). In this study, the state anxiety subscale (STAI-S) was used to measure the current anxiety level. The scale comprises 20 questions designed to measure state anxiety, and has an internal reliability coefficient of 0.87.

The Korean version of the State–Trait Anger Expression Inventory (STAXI) was used to measure current anger. High scores indicate more severe anger symptoms. As with STAI, STAXI is divided into subscales assessing state and traits (48, 49). We used the state anger subscale (STAXI-S) to measure current anger in this study. This questionnaire consists of 24 questions and has an internal reliability coefficient of 0.89.

### Statistical Analysis

Independent t-tests and Pearson’s correlations were used to compare continuous variables, and chi-square tests to compare categorical data. Non-parametric statistical tests such as the Kruskal–Wallis test or the Mann–Whitney U-test were used when it was not possible to assume regularity based on the Kolmogorov–Smirnov test. Stepwise multiple regression analysis was performed to test whether the interaction between early trauma on ELSQ (ET) and recent stressors on LES (RS) was a statistically significant predictor of CES-D, STAI-S, and STAXI-S scores. The dependent variables were CES-D, STAI-S, and STAXI-S scores. The independent variables were age, gender, ET, RS, and ET × RS. ET × RS was added as an independent variable to test the interaction of early trauma and recent stressors. The depressed group included subjects with a CES-D score ≥ 16; those with a score of ≤ 15 were classified into the non-depressed group. A regression analysis was performed on the depressed and non-depressed groups. SPSS Statistics ver. 25 was used for statistical analysis.

## Results

### Characteristics of the Study Subjects

In total, 170 subjects participated in the current study. Among them, 73 (42.9%) were men. There were no statistically significant sex differences in CES-D, STAI-S, or STAXI-S scores. The mean age of subjects was 41.1 ± 8.2 years. Age was not significantly correlated with CES-D, STAI-S, or STAXI-S scores (**Table 1**). There was no significant difference in age or sex between those with and without early trauma or between those with and without recent stressors. There was no significant association between the presence of early trauma and the occurrence of recent stressors.

Sixty-five (38.2%) subjects reported early traumatic experiences, and 40 (23.5%) reported recent stressful life events. Sixteen subjects (9.4%) were included in the ET-RS group, 49 (28.8%) in the ET-NRS group, 24 (14.1%) in the NET-RS group, and 81 subjects (47.6%) were included in the NET-NRS group (**Table 1**).

For all subjects, the mean ( ± SD) CES-D score was 11.7 ± 9.2, and the mean STAI-S and STAXI-S scores were 48.7 ± 7.7 and 12.7 ± 4.4, respectively (**Table 1**). Fifty-two subjects with a score ≥ 16 on the CES-D were assigned to the depressed group. CES-D was significantly correlated with both STAI-S (r = 0.658, *p <* 0.001) and STAXI-S (r = 0.564, *p <* 0.001) scores. The STAI-S and STAXI-S scores were also significantly correlated (r = 0.482, *p <* 0.001).

### Effects of Early Trauma and Recent Stressors on Depression

**Figure 1:**
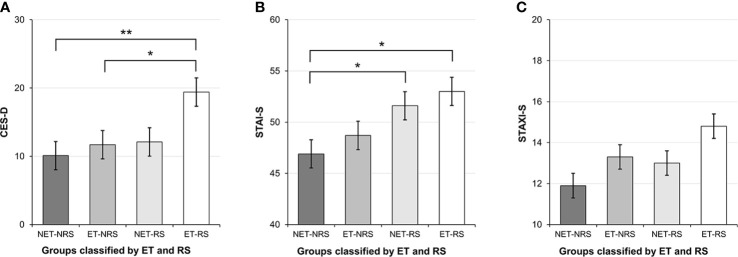
Depression, anxiety, and anger among subjects with or without early trauma and/or recent stressors. ET, presence of early trauma on the Early Life Stress Questionnaire; RS, presence of recent stressors on the Life Experience Survey; ET-RS, those with both early trauma and recent stressors; ET-NRS, those with early trauma but without recent stressors; NET-RS, those with recent stressors but without early trauma; NET-NRS, those with neither early trauma nor recent stressors; CES-D, Center for Epidemiological Studies-Depression Scale; STAI-S, state anxiety subscale of Korean version of State–Trait Anxiety Inventory; STAXI-S, The state anger subscale of the Korean version of the State–Trait Anger Expression Inventory. *p < 0.05, ^**^ *p <* 0.005. **(A)** Depression classified according to ET and RS. **(B)** Anxiety classified according to ET and RS. **(C)** Anger classified according to ET and RS.

The mean CES-D score of those who had experienced early trauma was significantly higher than that of subjects who had not (U = 2759.500, *p =* 0.036). Similarly, those who had experienced recent stressors had higher mean CES-D scores than those who had not (U = 2020.000, *p =* 0.033). The mean CES-D scores for the four groups were as follows: ET-RS, 19.4 ± 12.8; ET-NRS, 11.7 ± 8.2; NET-RS, 12.1 ± 10.3; NET-NRS, 10.1 ± 8.0 (**Table 1**). The difference among the groups was significant (H = 9.995, *p =* 0.019). Post hoc tests showed that the mean CES-D score of the ET-RS group was significantly higher than that of the ET-NRS (U = 245.500, *p =* 0.026) and NET-NRS groups (U = 332.500, *p =* 0.002) (**Figure 1A**). In the multiple regression models, the CES-D score was predicted by the interaction of early trauma and recent stressors (*R^2^* = 0.075, *p <* 0.001), but not by early trauma or recent stressors alone (**Table 2**). Similar results were found in the regression model after logarithmic transformation of CES-D.

**Table 2:** Effects of early trauma, recent stressors, and the early trauma–recent stressors interaction on depression, anxiety, and anger.

	*R^2^*	Age	Gender	ET	RS	ET x RS
CES-D score	0.075	–	–	–	–	β = 2.881^***^
STAI-S score	0.063	–	–	–	β = 4.560^**^	–
STAXI-S score	0.039	–	–	–	–	β = 0.994^*^

ET, presence of early trauma on the Early Life Stress Questionnaire; RS, presence of recent stressors on the Life Experience Survey; CES-D, Center for Epidemiological Studies-Depression Scale; STAI-S, state anxiety subscale of the Korean version of the State–Trait Anxiety Inventory; STAXI-S. state anger subscale of the Korean version of the State–Trait Anger Expression Inventory.

^*^p < 0.05, ^**^p < 0.005, ^***^p < 0.001.

### Effects of Early Trauma and Recent Stressors on Anxiety

Participants who had experienced recent stressors had higher average STAI-S scores than those who had not (t = 3.374, *p =* 0.001). Early trauma, however, had no significant effect on anxiety. The mean STAI-S scores in the four groups were as follows: ET-RS, 53.0 ± 7.6; ET-NRS, 48.7 ± 6.5; NET-RS, 51.6 ± 8.1; and NET-NRS, 46.9 ± 7.8 (**Table 1**). The difference among the groups was significant (H = 12.279, *p =* 0.006). Post hoc tests showed that the mean STAI-S scores of the ET-RS (U = 358.500, *p =* 0.005) and NET-RS groups (U = 647.500, *p =* 0.013) were significantly higher than that of the NET-NRS group (**Figure 1B**). In the multiple regression model, the only significant predictor of STAI-S scores was recent stressors (*R^2^* = 0.063, *p; =* 0.001) (**Table 2**). This result did not change with logarithmic transformation of STAI-S scores.

### Effects of Early Trauma and Recent Stressors on Anger

Participants who had experienced early trauma had higher STAXI-S scores than those who had not (U = 2826.000, *p =* 0.048). However, recent stressors showed no significant effect on anger. The mean STAXI-S scores in the four groups were as follows: ET-RS, 14.8 ± 6.0; ET-NRS, 13.3 ± 4.6; NET-RS, 13.0 ± 5.7; and NET-NRS, 11.9 ± 3.3 (**Table 1**). Overall, the groups did not differ significantly (**Figure 1C**), but a direct comparison of the ET-RS and NET-NRS groups revealed a significant difference (U = 440.000, *p =* 0.033). No other group differences were found. STAXI-S scores were predicted by the interaction of early trauma with recent stressors (*R^2^* = 0.039, *p = 0.010)*, but not by early trauma or recent stressors alone (**Table 2**). After logarithmic transformation of STAXI-S scores, similar results were found in the regression model.

### Predictors of Questionnaire Scores According to Depression Status

The CES-D scores of the depressed group were predicted by the interaction between early trauma and recent stressors (*R^2^* = 0.096, *p = 0.025*), but not by early trauma or recent stressors alone. No variables significantly predicted the STAI-S or STAXI-S scores in the depressed group. In contrast, the STAI-S scores of the non-depressed group were significantly predicted by recent stressors (*R^2^* = 0.065, *p = 0.005*). No other variables significantly predicted the CES-D or STAXI-S score in the non-depressed group.

## Discussion

The current study investigated the effect of early trauma, recent stressors, and their interaction on depression, anxiety, and anger in a community sample representing a broad age range. The results varied with the type of emotional disturbance examined. Depression and anger were mainly predicted by the interaction of early trauma and recent stressors, supporting our *a priori* hypothesis. However, contrary to our hypothesis, anxiety was predicted only by recent stressors and not by the early trauma–recent stressor interaction.

As expected, in the current study, both early trauma and recent stressors were associated with greater depression. When subjects were divided into four groups based on the presence or absence of early trauma and recent stressors, those who reported both early trauma and recent stressors were more depressed than those who had experienced only early trauma. Furthermore, depressive symptoms were predicted only by the interaction between early trauma and later stressors. The current findings suggest that depressive mood may be affected by synergistic effects of past childhood trauma and a current stressful environment.

Our finding corresponds to the stress sensitization model of depression, which proposes that the first depressive episode makes the individual more prone to subsequent depressive episodes following stressors (50, 51). Recently, stress itself has been suggested to produce sensitization for depression. According to this sensitization model, people who experienced early trauma are more easily depressed when they experience later stressors (37). Those without early trauma can fend off depression even under currently stressful conditions, and those with early trauma experiences can avoid depression if they are not currently experiencing stress.

The current findings correspond to those of previous studies reporting synergistic effects of early trauma and later stressors on the development of depression in young populations (37, 38). However, one study reported that childhood adversity did not modify the effect of recent events on depression of people 55–85 years of age (26). These discrepancies in findings may be due to differences between populations. The effect of early traumatic events may diminish as people get older, due to the longer time since the event rather than to age itself. Post-traumatic stress disorder (PTSD) symptoms after trauma tend to decrease with time, although there are some exceptions to this. Psychiatric symptoms caused by trauma decrease over time in veterans (52), young adults (53), and children (54). In addition, memories of a trauma tend to be vary depending on when they are evaluated (55, 56); thus, it appears that traumatic memories can change significantly over time. As the mean age of participants in our study was 41 years, our results suggest that the interactive effects of early trauma and current stress on depression might persist through middle age.

In the current study, as expected, anxiety was affected by recent stressors; however, it was not affected by early trauma (14, 16). The absence of a relationship between early trauma and current anxiety was contrary to previous findings showing that childhood adversity is linked to anxiety in adults (20, 57, 58). This discrepancy may reflect differences among studies in the severity of childhood trauma. Most previous studies focused on more serious trauma, such as physical or sexual abuse, whereas ours included any childhood adversity reported on the ELSQ. In addition, compared to depression or anger, anxiety may be highly affected by the present situation. Moreover, adulthood anxiety in victims of early trauma has been reported to vary depending on the person’s resilience (59–61). Those with low resilience become sensitized to emotional difficulties by childhood adversity, whereas others may even achieve posttraumatic growth after similar experiences (62, 63).

Anger was predicted only by the interaction between early trauma and recent stressors. Anger, like depression, is likely to be provoked when people who experienced childhood trauma encounter subsequent negative life events. Experiences of childhood trauma might result in a character structure that makes it easier to feel angry in stressful situations (24).

Although statistical insignificance necessarily means no real effects, some of the results in this study lost significance when the participants were divided into the depressed and non-depressed groups. The synergistic effects of early trauma and recent stressors on depression were found only in the depressed group. In contrast, the effects of recent stressors on anxiety were more prominent in the non-depressed group. These findings suggest that the interaction effect between early trauma and recent stressors on depression may be stronger in depressed than non-depressed groups. Non-depressed people may respond to current stress with anxiety, regardless of past childhood traumatic experience.

Usually, the CES-D is used for psychopathological evaluation of symptoms of depressive disorder while the STAI-S and STAXI-S are concerned with emotional problems. Our results may be related to the different dimensions measured by the questionnaires. The psychopathological problems measured by the CES-D may be more affected by the synergistic interaction between early trauma and recent stressors, while the emotional responses measured by the STAI-S are more related to current stressful events. However, we found that the STAXI-S scores were also associated with the synergistic interaction between early trauma and recent stressors, similar to the CES-D which is psychopathological evaluation. These complex results may be due to the infeasibility of distinguishing between psychopathological and emotional aspects in many situations. Severe anxiety or anger may be not only problematic but also pathological by themselves.

Previous studies have shown the effects of the interaction between early trauma and recent stressors on depression or suicidality in younger groups (37–39). To the best of our knowledge, the current study is the first to explore the interaction effect between early trauma and recent stressors on negative emotions such as anxiety or anger in a community sample with a wide age range.

The current study found that the effects of early and recent stressors and their interaction varied with the type of emotional disturbance considered. This finding may have some clinical implications. When individuals with early trauma encounter new stressful events, special clinical attention should be paid to the development or aggravation of depression or anger. For the management of anxiety, focusing on and coping with current stressful events rather than early experiences would be more helpful.

This study has several limitations. First, the number of subjects, especially of individuals who experienced recent stressors, was small. Thus, the impact of recent stressors may have been underestimated. Besides, convenience sampling was used to recruit the subjects. Thus, the generalizability of the result may be limited. Second, given that the information about early and recent stressors was obtained only by self-report, the data may be subject to recall bias or selective reporting and attempts to hide shameful or painful memories. Third, PTSD disease group was not analyzed separately. However, there were no subjects who reported diagnosis of PTSD at the time of the investigation. Besides, traumatic experiences do not always lead to PTSD (64). Especially, the ELSQ covers mild as well as severe traumatic experiences. The possibility of the recall bias of the early childhood experience would also be considered. Fourth, as this study was cross sectional, it could not show causal relationships or the mechanism underlying stressor interactions and their effects on emotions. Fifth, because stressful events were measured only in terms of their presence or absence, differences among individuals in the nature and severity of those experiences could not be considered. A future cohort study examining the detailed nature of such events in a large population may be needed to confirm the interaction effects of early and recent stressors on emotions.

In conclusion, our results showed a synergistic interaction between early trauma and recent stressors on current depression and anger. Anxiety, however, was affected mainly by recent stressors in the present study. Clinicians should be aware that clients’ emotional difficulties may depend on when they experienced traumatic or stressful experiences.

## Data Availability Statement

The raw data supporting the conclusions of this article will be made available by the authors, without undue reservation.

## Ethics Statement

The studies involving human participants were reviewed and approved by institutional review board of Gachon University of Medicine and Science. The patients/participants provided their written informed consent to participate in this study.

## Author Contributions

Conceptualization: SK, S-JC. Data curation: SK, S-JC, YL, JL, BS. Formal analysis: SJ, BS. Funding acquisition: SK. Investigation: SK, BS. Methodology: SK, S-JC, YL, JL, BS. Project administration: SK. Resources: SK, S-JC, YL. Supervision: SK. Software: YL, BS. Validation: SJ, JL. Visualization: BS. Writing—original draft: BS. Writing—review and editing: SJ, SK.

## Funding

This research was supported by the Brain Research Program through the National Research Foundation of Korea, funded by the Ministry of Science, ICT & Future Planning (No. 2016M3C7A1904336) and National Research Foundation of Korea (NRF) grant funded by the Korean government (MEST) (No. 2016R1A2B4011561).

## Conflict of Interest

The authors declare that the research was conducted in the absence of any commercial or financial relationships that could be construed as a potential conflict of interest.
